# A robust and validated integrated prognostic index for defining risk groups in adult acute lymphoblastic leukemia: an EWALL collaborative study^∗^^†^

**DOI:** 10.1182/bloodadvances.2023011661

**Published:** 2023-12-21

**Authors:** Amir Enshaei, Melvin Joy, Ellie Butler, Amy A. Kirkwood, Monica Messina, Chiara Pavoni, Mireia Morgades, Christine J. Harrison, Robin Foà, Josep-Maria Ribera, Sabina Chiaretti, Renato Bassan, Adele K. Fielding, Anthony V. Moorman

**Affiliations:** 1 Leukaemia Research Cytogenetics Group, Translational and Clinical Research Institute, Newcastle University, Newcastle upon Tyne, UK; 2 Cancer Research UK & UCL Cancer Trials Centre, UCL Cancer Institute, University College London, London, UK; 3 Gruppo Italiano Malattie Ematologiche dell'Adulto Foundation, Rome, Italy; 4 Unit of Hematology, Azienda Socio-Sanitaria Territoriale Papa Giovanni XXIII, Bergamo, Italy; 5 Clinical Hematology Department, ICO-Hospital Germans Trias I Pujol, Josep Carreras Research Institute, Badalona, Spain; 6 Hematology Department of Translational and Precision Medicine, Sapienza University, Rome, Italy; 7 Complex Operative Unit of Hematology, dell'Angelo Hospital and Santissimi Giovanni and Paolo Hospital, Mestre and Venice, Venezia-Mestre, Italy; 8 UCL Cancer Institute, London, United Kingdom

## Abstract

Risk stratification is crucial to the successful treatment of acute lymphoblastic leukemia (ALL). Although numerous risk factors have been identified, an optimal prognostic model for integrating variables has not been developed. We used individual patient data from 4 contemporary academic national clinical trials, UKALL14, NILG-ALL10/07, GIMEMA-LAL1913, and PETHEMA-ALL-HR2011, to generate and validate the European Working Group for Adult ALL prognostic index (EWALL-PI), which is based on white blood cell count, genetics, and end of induction minimal residual disease (MRD). Individual patient risk scores were calculated for 778 patients aged 15 to 67 years in complete remission using the validated UKALL-PI formula, applying minor modifications to reflect differences between pediatric and adult ALL. Per-trial analysis revealed that EWALL-PI correlated with relapse and death. Regression analysis revealed that each unit increase in EWALL-PI increased the risk of relapse or death by ∼30% with no evidence of heterogeneity across trials or patient subgroups. EWALL-PI–defined risk models outperformed the stratification algorithms used by each trial. Threshold analysis revealed an EWALL-PI threshold that divided patients with B cell and T cell into standard (EWALL-PI <2.50) and high (EWALL-PI ≥2.50) risk groups, respectively. Per-trial analysis showed that patients at high risk had a significantly increased relapse rate and inferior survival compared with patients with standard risk (subdistribution hazard ratio for relapse, ranged from 1.85 to 3.28; hazard ratio for death, 1.73 to 3.03). Subgroup analysis confirmed the robustness of these risk groups by sex, age, white blood cell count, and lineage. In conclusion, we validated an integrated risk model across 4 independent adult ALL clinical trials, demonstrating its utility defining clinically relevant risk groups.

## Key Points

• Integrating key risk factors (genetics, white blood cell count, and minimal residual disease) predicts outcome more accurately than using traditional risk factors independently.
• The EWALL-PI strongly correlates with relapse and death and defines clinically relevant risk groups in adult ALL.

## Visual Abstract

**Figure:**
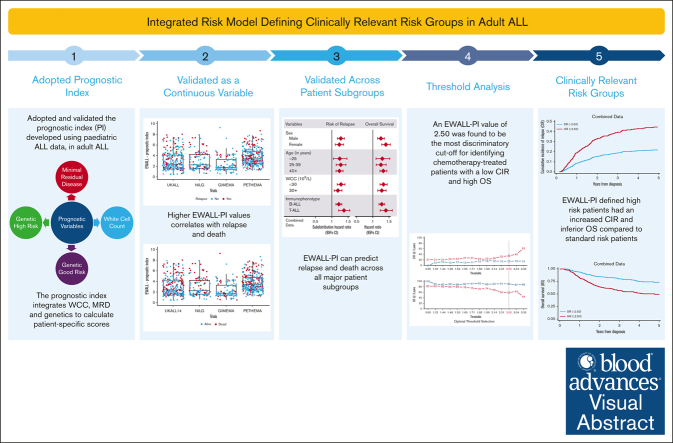

## Introduction

Accurate stratification according to the risk of treatment failure is vital for effective patient management of acute lymphoblastic leukemia (ALL) and improving outcome. Age is the single most common feature but also one of the most unsatisfactory ways to assign therapeutic pathways in ALL. A profusion of genomic studies over the past decade has identified numerous recurrent genetic abnormalities. The prognostic or predictive value of these potential biomarkers has yet to be fully determined and strategies for integrating them with other biomarkers are not well developed.

The most common method of stratification in ALL is to assign patients to treatment pathways based on several binary risk factors, applied independently of each other. These include age, white blood cell count (WCC) at presentation (>30 × 10^9^/L for B-cell ALL [B-ALL] and >100 × 10^9^/L for T-cell ALL [T-ALL]), presence or absence of minimal residual disease (MRD) at protocol-relevant time points, and presence or absence of high-risk genetic abnormalities (HR-GEN). However, dichotomizing continuous variables in this way both reduces their predictive power and predetermines the size of the risk groups. Furthermore, different national study groups and trials do not necessarily choose the same variables or thresholds. Only 2 previous studies have developed integrated prognostic models in adult ALL.^1^^,^^2^ Neither study considered MRD, and both lacked external validation cohorts. We have recently developed and validated an integrated prognostic index (PI), called UKALL-PI, using childhood, adolescent, and young adult patient cohorts that leverages the power of continuous data and provides a more flexible mechanism for defining risk groups.^3^ The PI integrates WCC, MRD, and genetics to calculate patient-specific scores.

UKALL-PI is age-agnostic, so we hypothesized that it could be applied to adult ALL cohorts. In this study, we validated the UKALL-PI using 4 independent contemporary adult ALL trial cohorts and demonstrate its utility to define risk groups that could be used to assign patients to treatment pathways. Furthermore, we demonstrate that an integrated PI is a superior method for defining risk groups in adult ALL compared with traditional systems.

## Patient cohorts and methods

We collected individual patient data from 4 clinical trials: UKALL14,^4^^,^^5^ NILG-ALL10/07,^6^ GIMEMA-LAL1913,^7^ and PETHEMA-ALL-HR2011.^8^ Patients provided written informed consent to trial treatment and correlative science studies according to the Declaration of Helsinki. Each study recruited patients with newly diagnosed ALL aged 15 to 67 years (supplemental Table 1). UKALL14, NILG-ALL10/07, and GIMEMA-LAL1913 used conventional eligibility criteria, but PETHEMA-ALL-HR2011 only recruited patients with high-risk features (supplemental Table 1). Treatment schedules differed, but each protocol comprised an induction phase lasting 5 to 10 weeks, ending with bone marrow MRD assessment, hereafter referred to as end of induction (EOI) MRD (supplemental Table 1). Postinduction therapy, including allogeneic stem cell transplant (allo-SCT), was determined by EOI MRD status, genetics, age, and WCC. However, each protocol applied different criteria and thresholds, so the proportion of patients with high-risk ALL and allo-SCT rate varied between trials (supplemental Table 1). The level of genetic screening differed between the 4 cohorts both in terms of prospective standard-of-care testing and retrospective research screening (supplemental Table 2). However, all cases in this study were classified into standard genetic/genomic subtypes as per the definition described for UKALL14.^9^

**Figure 1.**
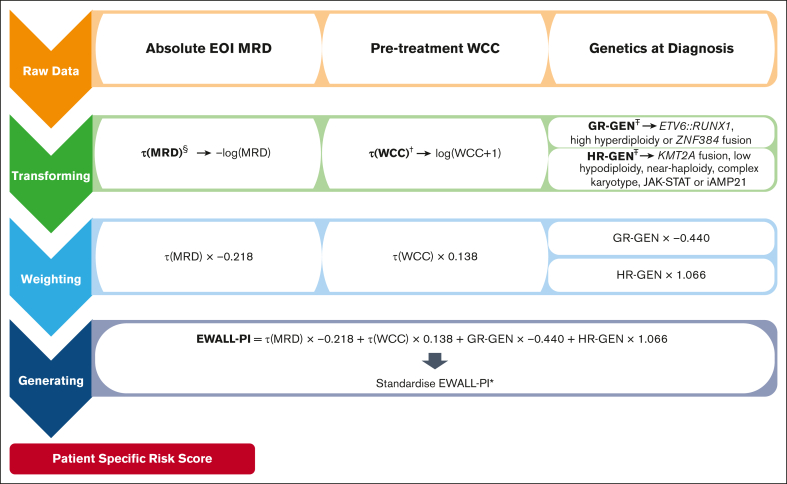
**Flow diagram describing the method for calculating the EWALL-PI for a patient.**^§^τ(MRD) was defined as the negative natural log of the absolute end of induction MRD level with undetectable MRD and MRD outside the quantitative range being assigned values of 1 × 10^–6^ and 1 × 10^–5^ respectively, whereas values ≥1 were rounded down to 0.99999. ^†^For this study, τ(WCC) was defined as log(WCC + 1) rather than log(WCC) + 1 to avoid negative numbers resulting from WCC values below 0.4 × 10^9^/L. ^Ŧ^GR-GEN and HR-GEN is coded as “1” if any of the listed genetic abnormalities is present, else “0.” ^∗^The EWALL-PI is standardized to make it range from 0 to 10, as follows: EWALL-PI = ([actual EWALL-PI − EWALL-PI minimum]/[EWALL-PI maximum − EWALL-PI minimum]) × 10; where EWALL-PI minimum = −3.6381537 and EWALL-PI maximum = 2.0882617. The minimum and maximum value of EWALL-PI was derived based on existing data sets. High hyperdiploidy, 51 to 67 chromosomes; low hypodiploidy, 30 to 39 chromosomes, including masked low hypodiploidy with 60 to 78 chromosomes; near-haploidy, <30 chromosomes; complex karyotype, ≥5 chromosomal abnormalities detected by karyotype analysis excluding established ploidy subgroups; JAK-STAT, *IGH::CRLF2*, *P2RY8::CRLF2*, *JAK2* fusions.

European Working Group for Adult ALL prognostic index (EWALL-PI) scores were calculated using the previously defined formula, applying minor modifications to reflect differences between pediatric and adult ALL (Figure 1): (1) the list of HR-GEN and good-risk genetic abnormalities (GR-GEN) was extended to include complex karyotype/JAK-STAT abnormalities and *ZNF384* fusions, respectively^3^; and (2) τ(WCC) was defined as log(WCC + 1) rather than log(WCC) + 1 to avoid negative numbers resulting from WCC values below 0.4 × 10^9^/L. JAK-STAT abnormalities were defined as *IGH::CRLF2*, *P2RY8::CRLF2* and *JAK2* fusions. Patients with *BCR::ABL1* fusion were not included in the original development of UKALL-PI,^3^ because they receive targeted therapy and often have MRD assessed by measuring *BCR::ABL1* fusion transcripts levels; so they were excluded from this study. Similarly, because EOI MRD is required to calculate EWALL-PI, patients whose ALL was not in complete remission (CR) at EOI were not included in this study.

Two end points were used in this study: overall survival (OS)—time to death, with censoring at date of last contact; and relapse rate (RR), assessed in a competing risks framework—time to relapse, with censoring at date of death in remission or last contact and death without relapse as competing event. Kaplan-Meier methods were used to estimate survival rates at 3 years. Subdistribution hazard ratios for each unit increase in PI were estimated using univariate and multivariable Fine-Gray competing risk model for relapse and hazard ratios from Cox model for death. χ^2^ test or Fisher exact test were used to compare proportions and Mann-Whiney *U* tests to compare medians across groups and assess distributions. Shapiro-Wilk test was used to assess normality. We used standard principles and methods to validate the PI.^10^ Models were assessed using Harrell's concordance index (C-index) and calibrated by comparing the predicted and observed event probability. To examine subdistribution hazard ratios/hazard ratios across different patient subgroups, we used forest plots and the test of heterogeneity. To identify the optimal thresholds for standard and high-risk groups, we used Youden's index^11^ and threshold analysis. *P* values <.05 were considered significant. All the analyses were performed using Intercooled Stata 18.0 (StataCorp, College Station, TX) and R version 4.2.3.^12^ R packages ggplot2 (3.4.0), ggforestplot (0.1.0), and forestplot (3.1.1) were used for data visualization.

## Results

### Validation of the EWALL-PI in adult ALL

We calculated patient specific EWALL-PI scores for a total of 778 adult patients diagnosed with ALL from 4 modern MRD-driven clinical trial cohorts run over a similar time period: UKALL14 (n = 253), NILG-ALL10/0712 (n = 109), GIMEMA-LAL1913 (n = 108), and PETHEMA-ALL-HR2011 (n = 308). A per-trial comparison revealed that the characteristics of patients for whom a PI could be calculated were representative of the total eligible cohort within each trial (supplemental Table 2). For GIMEMA-LAL1913, the PI cohort had a slight overrepresentation of patients aged <25 years (31% vs 23%; *P* = .05) and underrepresentation of patients with HR-GEN (15% vs 8%; *P* = .04) compared with the total eligible cohort (supplemental Table 2). Twenty-five patients in the GIMEMA-LAL1913 cohort had HR-GEN, but 16 (64%) did not have an EOI MRD result, so a PI could not be calculated.

**Figure 2.**
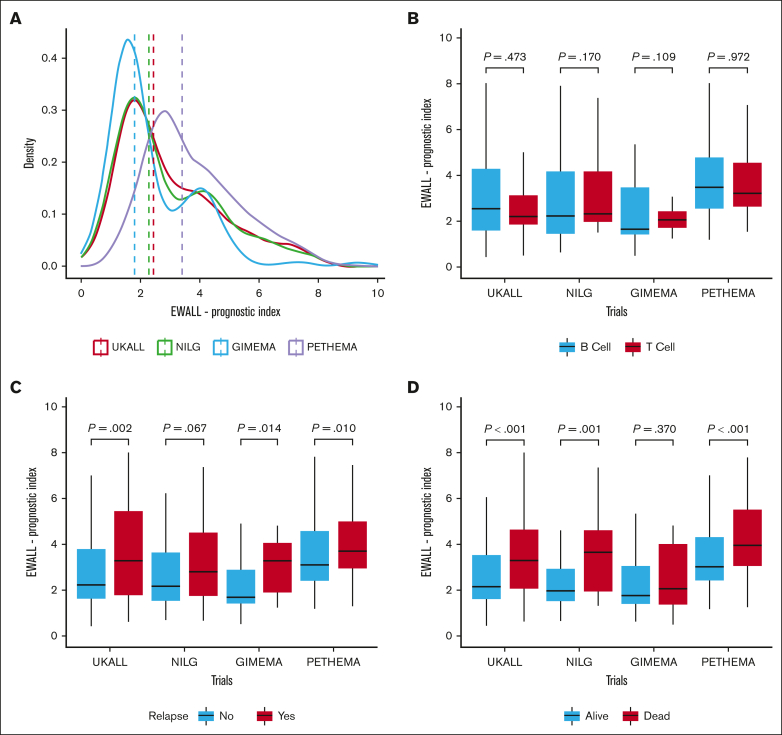
**Distribution of EWALL-PI by trial and within each trial immunophenotype, relapse, and death.** (A) The dotted vertical line shows the median EWALL-PI for each trial. The UKALL14 distribution was significantly different to GIMEMA-LAL1913 (*P* < .001) and PETHEMA-ALL-HR2011 (*P* < .001) but not NILG-ALL10/07 (*P* = .73) (*P* values from a Kolmogorov–Smirnov test for equality of distribution). (B) There was no difference between the EWALL-PI distributions for patients with B-ALL and T-ALL within each trial. (C) The median EWALL-PI values for patients treated on UKALL14, GIMEMA-LAL1913, and PETHEMA-ALL-HR2011 who relapse was significantly greater than those patients who did not relapse. (D) Similarly, the median EWALL-PI values for patients treated on UKALL14, NILG-ALL10/07, and PETHEMA-ALL-HR2011 who died was significantly greater than those patients who did not die. The *P* values reported in boxes B, C and D are from a Mann-Whitney *U* test because the EWALL-PI was not normally distributed using a Shapiro-Wilk Test for normality.

**Figure 3.**
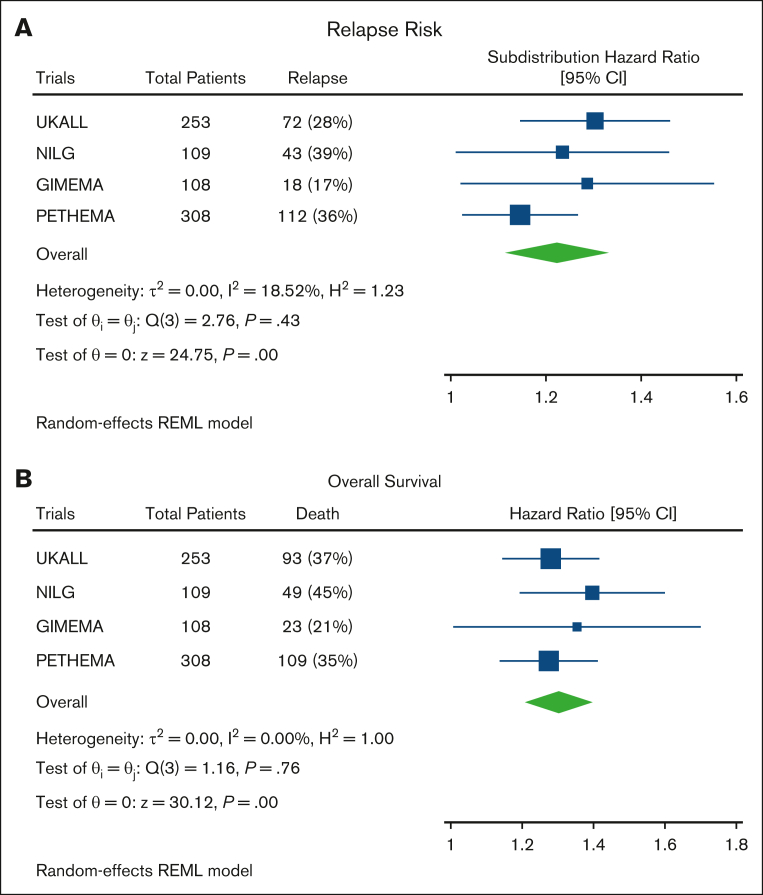
**Forest plots and tests of heterogeneity comparing risk of relapse and death across the 4 different trials.** (A) Subdistribution hazard ratios from Fine-Gray competing risk models for risk of relapse. (B) Hazard ratios from Cox regression analysis for risk of death.

The EWALL-PI distributions differed between the studies (Figure 2A), reflecting the distinct cohort characteristics, including MRD time point and proportion of HR-GEN (supplemental Table 2). Patients in the PETHEMA-ALL-HR2011 study had higher presenting WCC and, due to the earlier time point of measurement, higher MRD levels. Hence patients in PETHEMA-ALL-HR2011 had higher EWALL-PI scores illustrated by a higher median and right-shifted distribution (Figure 2A). In contrast, the GIMEMA-LAL1913 distribution was shifted to the left (Figure 2A), reflecting overrepresentation of younger patients and underrepresentation of patients with HR-GEN (supplemental Table 1). Within each trial, there was no difference in distribution of EWALL-PI by immunophenotype (Figure 2B). EWALL-PI was associated with outcome in all 4 trials (Figure 2C-D). Patients who relapsed and/or died had, on average, higher EWALL-PI scores. Univariate Cox regression analysis revealed that, on average, each unit increase in EWALL-PI score was associated with a 24% (range, 15%-30%) increased risk of relapse and a 32% (range, 27%-40%) increased risk of death with no evidence of heterogeneity between the 4 trials (Figure 3). Subdividing the combined cohort into 5 equally sized groups, according to EWALL-PI, revealed a correlation between higher EWALL-PI values and inferior outcome (supplemental Figure 1). Multivariable analysis revealed that the magnitude of the hazard ratio and the significance level were retained when age was added to the model as a continuous variable (supplemental Table 3).

Each trial used a different algorithm based on multiple unitary risk factors to define treatment risk groups (supplemental Table 1). To evaluate the original algorithms against the novel EWALL-PI model, we compared the fit of the models using Harrell's C-index. The EWALL-PI model using a continuous variable outperformed all the original risk models for both RR and OS (supplemental Table 4), confirming the benefit of selecting, weighting, and integrating risk factors. We calibrated the EWALL-PI Cox model by predicting survival probabilities in NILG-ALL10/07, GIMEMA-LAL1913, and PETHEMA-ALL-HR2011 compared with UKALL14 and observed no significant deviations (*P* = .91; *P* = .61; and *P* = .19, respectively) (supplemental Figure 2). Examining the coefficients of the different EWALL-PI elements across the 4 trials showed a high level of robustness for MRD and WCC (supplemental Figure 3). The variation observed for genetics was expected given the differences in genetic screening and rarity of GR-GEN in adult ALL. Subgroup analysis confirmed that EWALL-PI can predict relapse and death outcome across all major patient subgroups (supplemental Figure 4).

### Application of the EWALL-PI in a clinical trial setting

EWALL-PI scores correlate with survival and so can be used to define risk groups. Because the majority of adult ALL protocols assign patients to risk groups at the EOI, we sought to identify 2 exemplar risk groups, standard risk (SR) and high risk (HR), using EWALL-PI that could provide better prediction of risk than traditional algorithms. The 4 trials in this study used different algorithms to define risk groups. (supplemental Table 1). The UKALL14 cohort had undergone extensive screening for the genetic abnormalities, so we opted to use this population as the discovery cohort. We focused on patients who received chemotherapy only because this provided a uniformly treated population without the competing risk of transplant-related mortality. Because patients with HR ALL treated on UKALL14 were eligible for allo-SCT, the chemotherapy-only cohort had a preponderance of SR features (supplemental Table 6). There were 34 patients classified as HR by UKALL14 criteria who did not proceed to allo-SCT due to donor availability (supplemental Table 6). Although 13 of these patients relapsed, the time to relapse ranged from 189 to 2381 days; longer than median and mean time to transplant in UKALL14, which was 158 and 172 days, respectively.

**Figure 4.**
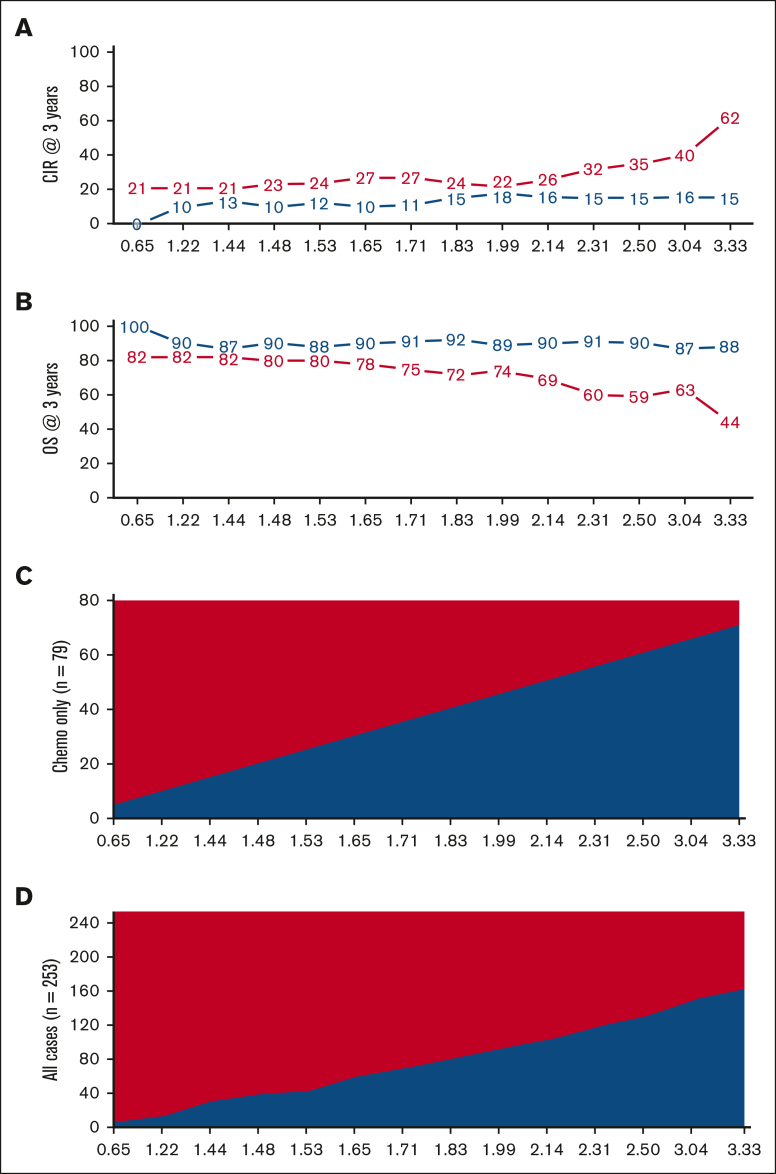
**Optimal threshold selection in the UKALL14 cohort.** To determine the optimal EWALL-PI threshold for defining risk groups, we assessed 14 selected thresholds using a cohort of 79 patients treated on UKALL14 with chemotherapy only. The cumulative incidence of relapse (CIR [A]) and OS rates (B) were calculated for patients above (red) and below (blue) each threshold. (C-D) show the proportion of patients with EWALL-PI scores above (red) and below (blue) each of the selected EWALL-PI thresholds. Threshold analysis was performed by sorting the PI, dividing the cohort into bins comprising ∼5 cases (∼6% cohort) and sequentially testing each threshold until the exemplar clinical criteria were met. The most discriminatory threshold (ie, the one with the maximum difference between CIR and OS rates) was 2.50, which divided the chemotherapy cohort 75%/25% but the whole UKALL14 cohort 52%/48%. Patients with a EWALL-PI score <2.50 had a CIR at 3 years of 15% vs 35% for patients with a score ≥2.50 (*P* < .001). The equivalent OS rates at 3 years were 90% vs 59% (*P* < .001).

**Figure 5.**
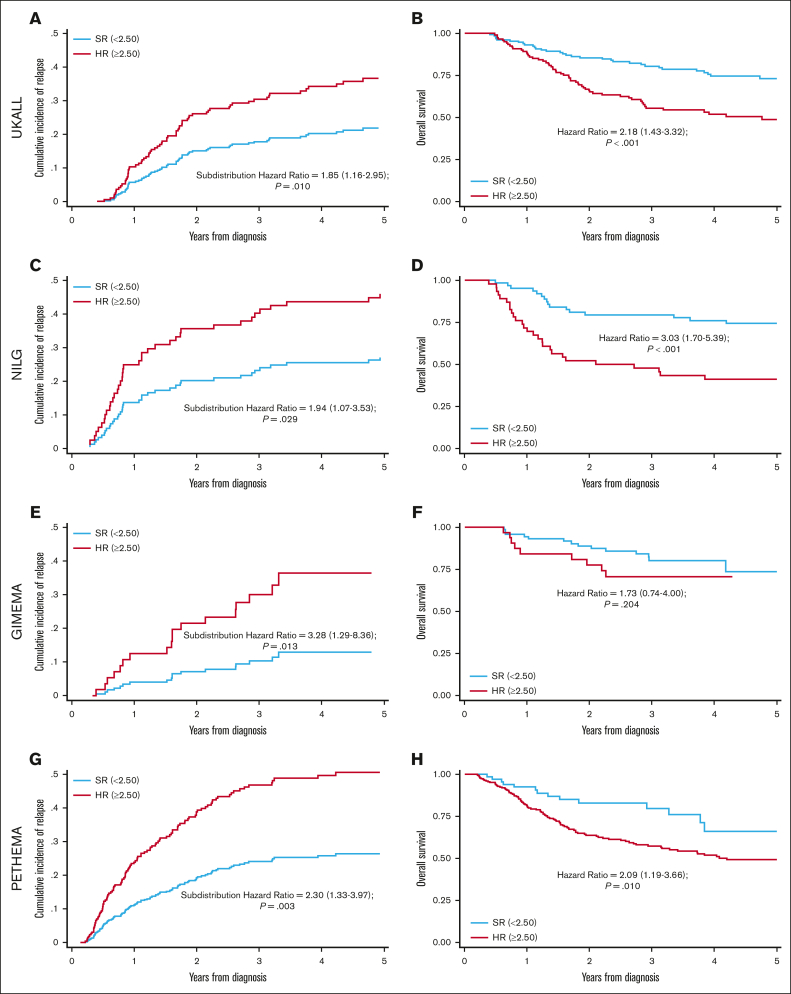
**Validation of EWALL-PI–defined risk groups across the 4 trials.** UKALL14 (A-B), NILG-ALL10/07 (C-D), GIMEMA-LAL1913 (E-F) and PETHEMA-ALL-HR2011 (G-H). Panels A,C,E,G show Kaplan-Meier plots for CIR and the subdistribution hazard ratio for increased risk of relapse for patients at HR vs patients at SR. Panels B,D,F,H show the hazard ratio for OS. Patients with a EWALL-PI <2.50 were assigned to the SR group (blue lines) whereas patients at HR had EWALL-PI ≥2.50 (red lines).

**Figure 6.**
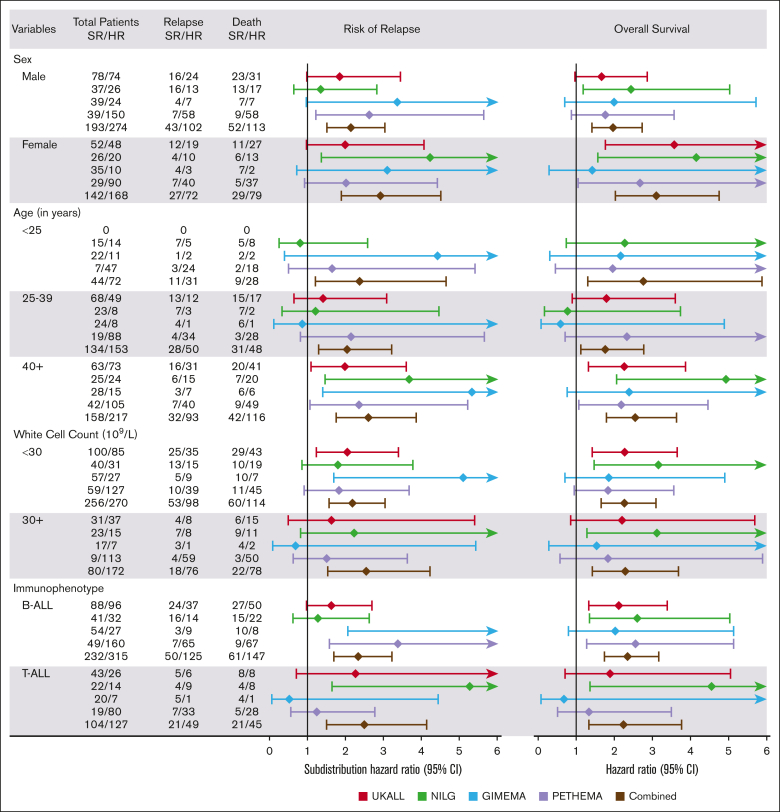
**Forest plot showing the subdistribution hazard ratios for risk of relapse derived from Fine-Gray competing risk model and hazard ratios for death derived from Cox regression analysis, comparing patients in the EWALL-PI–defined HR and SR groups stratified by key patient subgroups within each trial and using a combined data set.**

Threshold analysis and Youden's index determined that an EWALL-PI value of 2.50 was the most discriminatory cutoff for identifying chemotherapy-treated patients with a low cumulative incidence of relapse (CIR) and high OS at 3 years (Figure 4). This threshold was validated across all 4 trials in terms of CIR and across all studies except GIMEMA for OS (Figure 5). Because we used patients treated in UKALL14 by chemotherapy-only to determine the threshold, we performed a subgroup analysis by treatment pathway (chemotherapy vs allo-SCT) to confirm that the threshold applied to all patients treated on UKALL14 (supplemental Figure 5). As expected, patients in the SR group were typically associated with younger age, lower WCC, and lower allo-SCT rates (Table 1). None of the patients assigned to the SR group had EOI MRD ≥0.01% or HR-GEN, indicating that the weights applied to these variables in the EWALL-PI model were always sufficient to generate a score ≥2.50. Across all the trials, patients assigned to the HR group had a significantly increased risk of relapse and/or death compared with those assigned to the SR group (hazard ratio ranging from 1.73 to 3.28) (Figure 5). Subgroup analysis revealed that this prognostic effect was robust across major patient subgroups both within each trial and when the cohorts were combined (Figure 6).

**Table 1.** **Demographic, clinical, genetic, and outcome features of patients assigned to the EWALL-PI–defined risk groups in each of the 4 trials**

Variables	UKALL14			NILG-ALL10/07			GIMEMA-LAL1913			PETHEMA-ALL-HR2011		
	SR	HR	*P* value	SR	HR	*P* value	SR	HR	*P* value	SR	HR	*P* value
Total cases	131	122		63	46		74	34		68	240	
**Sex**												
Female	52 (39.7)	48 (39)	.62	26 (41)	20 (43)	.82	35 (47)	10 (29)	.08	29 (43)	90 (38)	.44
Male	78 (59.5)	74 (61)		37 (59)	26 (57)		39 (53)	24 (71)		39 (57)	150 (63)	
Intersex	1 (0.8)	0 (0)		-	-		-	-		-	-	
**Age, median (range), y**	39 (25-65)	44 (25-63)	.06	36 (17-61)	42 (18-67)	.69	33 (18-65)	36 (18-65)	.70	45 (15-60)	37 (15-60)	.001
<25	-	-	.07	15 (24)	14 (30)	.03	22 (30)	11 (32)	.80	7 (10)	47 (20)	.05
25-39	68 (52)	49 (40)		23 (37)	8 (17)		24 (32)	8 (24)		19 (28)	88 (37)	
40-59	57 (44)	60 (49)		24 (38)	18 (39)		21 (28)	12 (35)		41 (60)	102 (43)	
60+	6 (5)	13 (11)		1 (2)	6 (13)		7 (9)	3 (9)		1 (1)	3 (1)	
**WCC, median (range), 10^9^/L**	8.2 (0.1-338.8)	12.2 (0.4-583.1)	.03	9.4 (0.4-268.0)	15.7 (1.7-1021.4)	.23	6.0 (0.3-209.5)	8.1 (0.4-347.3)	.99	4.0 (0.2-244.0)	23.2 (0.6-638.0)	<.001
<30	100 (76)	85 (70)	.28	40 (63)	31 (67)	.70	57 (77)	27 (79)	.16	59 (87)	127 (53)	<.001
30-49	7 (5)	12 (10)		5 (8)	4 (9)		6 (8)	0 (0)		2 (3)	28 (12)	
50-99	13 (10)	9 (7)		7 (11)	2 (4)		7 (9)	2 (6)		2 (3)	30 (13)	
≥100	11 (8)	16 (13)		11 (17)	9 (20)		4 (5)	5 (15)		5 (7)	55 (23)	
**Immunophenotype**												
B cell	88 (67)	96 (79)	.04	41 (65)	32 (70)	.62	54 (73)	27 (79)	.47	49 (72)	160 (67)	.40
T cell	43 (33)	26 (21)		22 (35)	14 (30)		20 (27)	7 (21)		19 (28)	80 (33)	
**MRD**												
Negative (<0.01%)	131 (100)	60 (49)	<.001	63 (100)	14 (30)	<.001	74 (100)	11 (32)	<.001	68 (100)	117 (49)	<.001
Positive (≥0.01%)	0 (0)	62 (51)		0 (0)	32 (70)		0 (0)	23 (68)		0 (0)	123 (51)	
**Genetic risk group**												
Good	13 (10)	6 (5)	.13	3 (5)	0 (0)	.13	9 (12)	2 (6)	.50	5 (7)	4 (2)	.03
High	0 (0)	66 (54)	<.001	0 (0)	17 (37)	<.001	0 (0)	9 (26)	<.001	0 (0)	37 (15)	<.001
**Outcome**∗												
Relapse	29 (22)	43 (35)	.02	20 (32)	23 (50)	.05	8 (11)	10 (29)	.02	14 (21)	98 (41)	.002
Death	35 (27)	58 (48)	<.001	19 (30)	30 (65)	<.001	14 (19)	9 (26)	.37	14 (21)	95 (40)	.004
Transplant	72 (55)	102 (84)	<.001	23 (37)	29 (63)	.01	17 (23)	12 (35)	.18	4 (6)	66 (28)	<.001
**Survival rates at 3 y**												
Cumulative incidence of relapse	16% (11-23)	32% (24-40)	.003	19% (11-30)	46% (31-59)	.007	12% (5-20)	28% (13-46)	.005	28% (16-42)	46% (39-53)	.001
OS	80% (72-86)	56% (46-64)	<.001	79% (67-87)	48% (33-61)	<.001	80% (68-88)	71% (51-84)	.20	80% (65-88)	57% (50-64)	.01

∗ *P* values comparing relapse, death and transplant are from a χ^2^ test which does not factor in censoring.

The EWALL-PI–defined risk groups produced higher C-index scores than trial-defined risk groups when RR was considered and, for UKALL14 and NILG-ALL10/07, when OS was considered (supplemental Table 4). Because the trial risk groups do not always align perfectly with whether the patients actually received an allo-SCT in first remission, we examined the C-index scores using allo-SCT received as an outcome measure. Again, the EWALL-PI–defined risk groups produced equivalent or higher C-index scores, confirming the superiority of EWALL-PI–defined risk group (supplemental Table 4). Given that both the original and EWALL-PI–defined risk groups used overlapping risk factors to stratify patients, it was not surprising that there was a strong association between the 2 schemas in UKALL14, NILG-ALL10/07, and PETHEMA-ALL-HR2011 (all *P* < .001) (supplemental Table 7). The lack of association observed in the GIMEMA-LAL1913 (*P* = .74) cohort is likely to be driven by the fact that a high proportion (64%) of patients with HR-GEN were lacking EOI MRD values and hence not included in the PI cohort (supplemental Table 2).

A total of 188 patients across the 4 trials were identified by EWALL-PI as HR but were treated as SR on the trial. These patients tended to have high CIR and low OS rates, highlighting the benefit of using an integrated risk model to define risk groups (supplemental Table 7). Interestingly, 130 patients were identified by EWALL-PI as SR but had been treated as HR on trial. These patients had outcomes equivalent to patients treated as SR (supplemental Table 7). Similar results were observed when we examined differences according to the postinduction treatment received rather than by risk group. Patients identified as HR by EWALL-PI but who did not receive an allo-SCT had very high relapse rates (>50%) especially in NILG-ALL10/07 and PETHEMA-ALL-HR2011 (supplemental Table 7). The reasons why these patients did not receive an allo-SCT in first remission will be varied and complicated. However, it clearly confirms the ability of the EWALL-PI to identify patients who require more than chemotherapy after induction. Conversely, the OS of patients defined as SR by EWALL-PI who received chemotherapy-only after induction was ≥75% at 3 years in all 4 trials (supplemental Table 7). The ratio of SR to HR varied between each trial in line with the different demographic and clinical features of each trial. The PETHEMA-ALL-HR2011 had the highest proportion of HR cases in keeping with its eligibility criteria (supplemental Tables 1 and 2). By combining UKALL14, NILG-ALL10/07, and GIMEMA-LAL1913, we estimate that the size of the SR and HR groups generated by using an EWALL-PI threshold of 2.50 will be 43%/57% overall, 42%/58% in B-ALL, and 45%/55% in T-ALL (Table 1).

## Discussion

Using individual patient data from 4 contemporary adult ALL clinical trials, we demonstrate that EWALL-PI provides a robust tool for predicting outcome in adult ALL. EWALL-PI is based on UKALL-PI, which was developed and validated using pediatric, adolescent, and young adult data sets.^3^ UKALL-PI is age-agnostic, so we were able to apply the same formula in adult ALL; only making minor modifications to reflect differences between pediatric and adult ALL. Importantly, these minor modifications did not alter the contribution of each factor to the PI (supplemental Figure 3) compared with that observed in the original pediatric, adolescent, and young adult data sets.^3^ The index applied in this study is referred to as EWALL-PI because it used data from national studies from 3 European countries participating in the EWALL consortium. Previous attempts to develop an integrated prognostic model in adult ALL have clearly demonstrated the superiority of this approach compared with using individual risk factors.^1^^,^^2^ However, neither study was able to validate the model using external cohorts and neither considered EOI MRD in the modeling process. Although both studies identified a HR group that had very poor outcome of OS <25% at 2 years, the outcome of the patients in the SR group was also relatively poor at ∼50%. Although the poor outcome of the SR groups is likely due to the absence of EOI MRD in the model, it reduces the clinical utility of the models.

EWALL-PI offers an innovative approach to risk stratification in adult ALL and has many advantages over previous algorithms. Crucially, it does not require the generation of any new data. Measuring presenting WCC, EOI MRD, and genetic testing are standard of care in adult ALL. Although the list of GR-GEN and HR-GEN abnormalities may be longer than that used in current risk stratification algorithms, none should pose a significant challenge nor the adoption of a nonstandard test. Manual calculation of UKALL-PI would be time consuming and error prone, but it will be simplified using preformatted Excel files or webpages that require the user to enter a minimal simple data. An example of such an Excel spreadsheet is provided in the supplemental Information (supplemental Table 8). The novelty and major strengthen of EWALL-PI are the integration and weighting of the risk factors and leveraging of continuous data. This methodology provides increased precision for predicting outcome, and EWALL-PI outperforms existing risk algorithms. Importantly, we have validated EWALL-PI robustly using 4 diverse adult ALL trials and demonstrated its applicability across major patient subgroups; including patients aged up to 60 to 65 years and patients with T-ALL. The applicability of EWALL-PI in patients aged >65 years and those with only extramedullary disease remains to be determined. Prospective evaluation of the EWALL-PI in a clinical trial setting would provide definitive proof of its superiority and applicability in adult ALL.

This retrospective study has the inherent and unavoidable limitation that the patients had already been treated. The use of different upfront risk algorithms meant the HR ALL groups were heterogenous in terms of demographics and different proportions of patients were assigned to allo-SCT. However, we demonstrated that patients identified as HR by EWALL-PI but treated as SR had inferior outcomes compared with patients identified by EWALL-PI as SR and treated as SR. All risk factors and algorithms require evaluation when novel therapies are introduced, and EWALL-PI is no exception. The use of new therapies (eg, blinatumomab) in frontline therapy for adult ALL will hopefully improve patient outcome. However, they may also influence the prognostic impact of existing risk factors, including MRD if given during induction therapy before MRD assessment. Future trials using immunotherapy will need to evaluate the performance of any risk algorithm (eg, EWALL-PI) to assess the characteristics and number of patients assigned to each risk group and the impact on outcome. Another limitation of EWALL-PI is that it is not applicable to all adult patients with ALL. Patients with *BCR::ABL1* fusion were excluded from the study because MRD measurement is via fusion transcript, and they receive targeted therapy. However, the methodology used to develop and validate EWALL-PI could readily be applied to this large and important subset of adult ALL, because EOI MRD is a crucial component of EWALL-PI patients whose ALL was not in CR at this time point. Currently no genomic driver of resistant disease has been identified, so an integrated approach (with a very early response time point) could be beneficial for identifying these cases.

The original UKALL-PI defined GR-GEN as *ETV6::RUNX1* and high hyperdiploidy, whereas HR-GEN abnormalities were iAMP21, *KMT2A* fusions, near-haploidy, low hypodiploidy, and *TCF3::HLF*. Numerous studies have reported the high-risk nature of *KMT2A* fusions and low hypodiploidy in adult ALL.^13^, ^14^, ^15^, ^16^, ^17^ Although *ETV6::RUNX1*, iAMP21, *TCF3::HLF*, and near-haploidy are very rare in adult ALL, the data that do exist suggest a similar prognostic effect as reported in pediatric ALL.^13^, ^14^, ^15^, ^16^, ^17^, ^18^, ^19^ Complex karyotype and JAK-STAT abnormalities (*CRLF2*-r/*JAK2*-r) were added to the HR-GEN list because they have been linked to a poor outcome in several previous adult ALL studies.^9^^,^^13^^,^^14^^,^^16^, ^17^, ^18^^,^^20^^,^^21^ Three independent studies have reported a favorable outcome for adult patients with *ZNF384* fusions, so we included these cases in the GR-GEN group.^9^^,^^22^^,^^23^ The impact of additional putative HR-GEN (eg, *IKZF1* deletion, IKZF1plus, and ABL-class fusions) in relation to EWALL-PI should be evaluated prospectively.

Screening for GR/HR-GEN abnormalities requires the application of multiple different techniques. It is important to note that the cohorts used in this study were not prospectively screened for all GR/HR-GEN abnormalities because many were not used for patient management at the time of diagnosis. Although retrospective screening was performed, it was not comprehensive. So, the proportion of GR-GEN and HR-GEN abnormalities varies across the 4 cohorts. Missing genetic information does not prevent the risk score being calculated. Such cases will be treated as lacking both GR-GEN and HR-GEN, and hence, the genetic variables will contribute “zero” to the final risk score. This variation between the cohorts can be viewed both as a limitation and a strength of the study. EWALL-PI is designed to weight both GR-GEN and HR-GEN, so undetected cases will shift risk scores toward the median, diluting the effect of the risk score. In contrast, the presence of missing data represents a real-world test. The fact that EWALL-PI was validated across all data sets, despite this variation, supports its generalizability and applicability. Going forward, the reliable detection of all GR-GEN and HR-GEN genetic abnormalities is paramount to optimizing the clinical use of EWALL-PI. A combination of standard-of-care tests, such as cytogenetics, FISH, SNP array, and RNA fusion panels, can be deployed to readily detect all the abnormalities needed to calculate EWALL-PI.^24^

The majority of adult ALL trials seek to divide patients into 2 risk groups with the higher-risk group being eligible for allo-SCT, whereas the patients at SR will receive chemotherapy. This decision is made at EOI and only applicable to those patients whose ALL is in CR. All the data required to compute EWALL-PI will be available at the EOI, including MRD and genetics. All the analysis presented in this study is based on patients whose ALL is in CR by EOI so can directly inform this process. The diversity of risk stratification algorithms applied in adult ALL prevents direct comparison of trial outcomes and is an impediment to designing multinational clinical trials, which require a single unified algorithm. One of the driving forces behind the study was to develop a risk stratification algorithm that could be applied in a multinational clinical trial setting. We acknowledge that the C-index for the categorical EWALL-PI model are modest, but it is important to note that they are as good as or better than existing algorithms (supplemental Table 4). Genetics, especially high-risk genetics, is a key factor when calculating the PI. As discussed above, genetic screening of these cohorts was incomplete, hence, it is likely that the EWALL-PI would perform better prospectively with contemporary standard-of-care genetic testing. We present data showing that EWALL-PI can be used to define SR (PI < 2.50) and HR (≥2.50) groups, which have significantly different outcomes across 4 independent trials. Importantly, this effect is retained across all major patient subgroups and means it can be applied to both B-ALL and T-ALL. This threshold will generate clinically useful groups that will be applicable to many studies. However, EWALL-PI is a continuous variable correlating directly with outcome, so a new threshold or thresholds can be selected to generate the number and size of risk groups required for a study.

In conclusion, we present a robust and independent validation of a flexible risk score in adult ALL. The ACCADEMIA study group members (UK National Cancer Research Institute Adult ALL Group, Gruppo Italiano Malattie Ematologiche dell’Adulto [GIMEMA], Programa de Estudio y Tratamiento de las Hemopatías Malignas [PETHEMA], and Hemato-Oncologie voor Volwassenen Nederland [HOVON]) will adopt EWALL-PI to risk stratify patients in the forthcoming ACCADEMIA trial.

Conflict-of-interest disclosure: The authors declare no competing financial interests.
