# Supplementary material for: A robust and validated integrated prognostic index for defining risk groups in adult acute lymphoblastic leukemia: an EWALL collaborative study
Source: Blood Adv. 2023 Dec 21;8(5):1155–66. doi: 10.1182/bloodadvances.2023011661 (PMC10910126; doi:10.1182/bloodadvances.2023011661)
Supplement: Supplemental Figures and Tables [file BLOODA_ADV-2023-011661-mmc1.pdf]

A

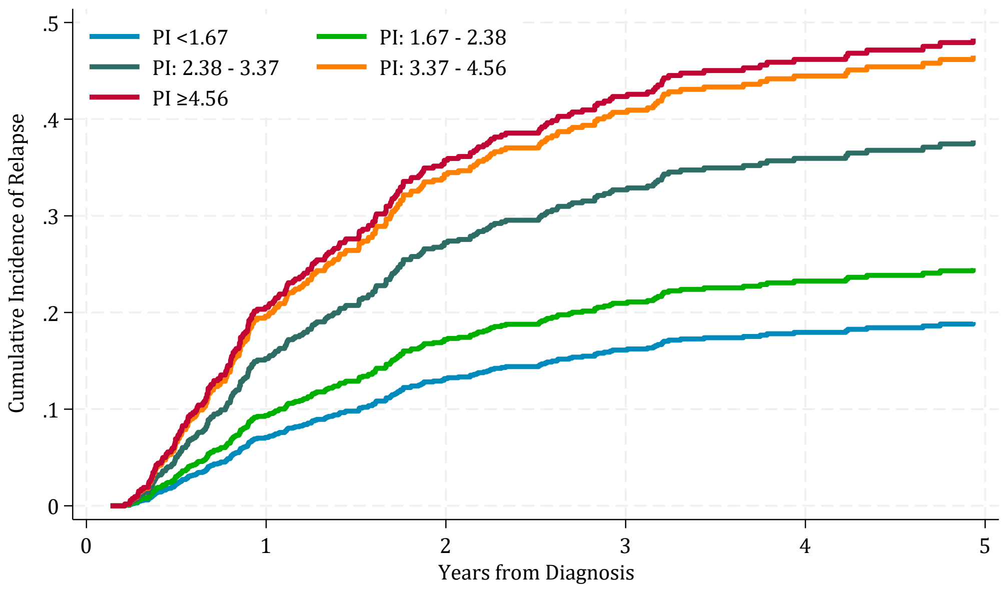

B

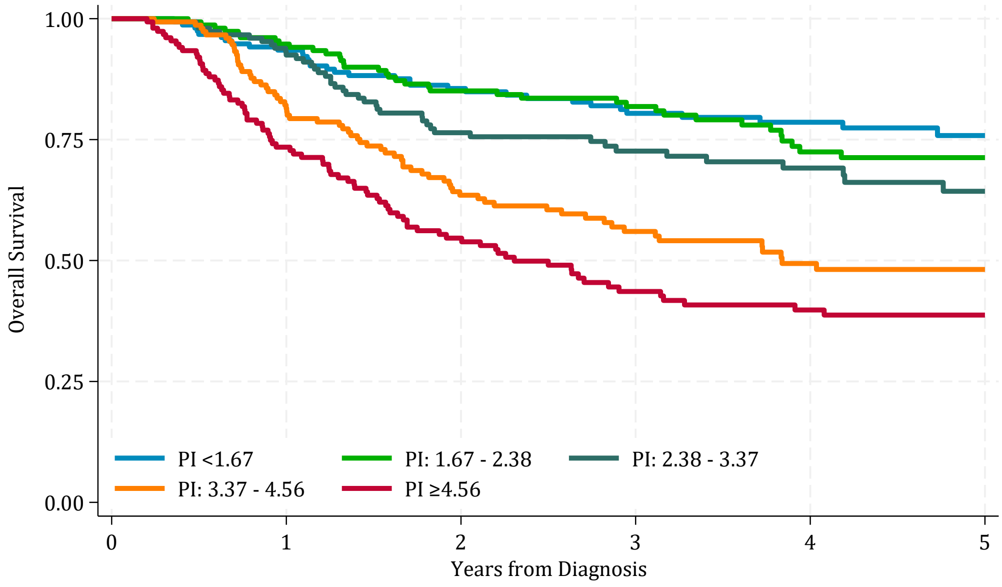

Supplementary Figure 1: Kaplan Meier plots demonstrating the increasing EWALL-PI scores correlate with increased risk of relapse (A) and decreased overall survival (B). Abbreviation: PI - prognostic index.

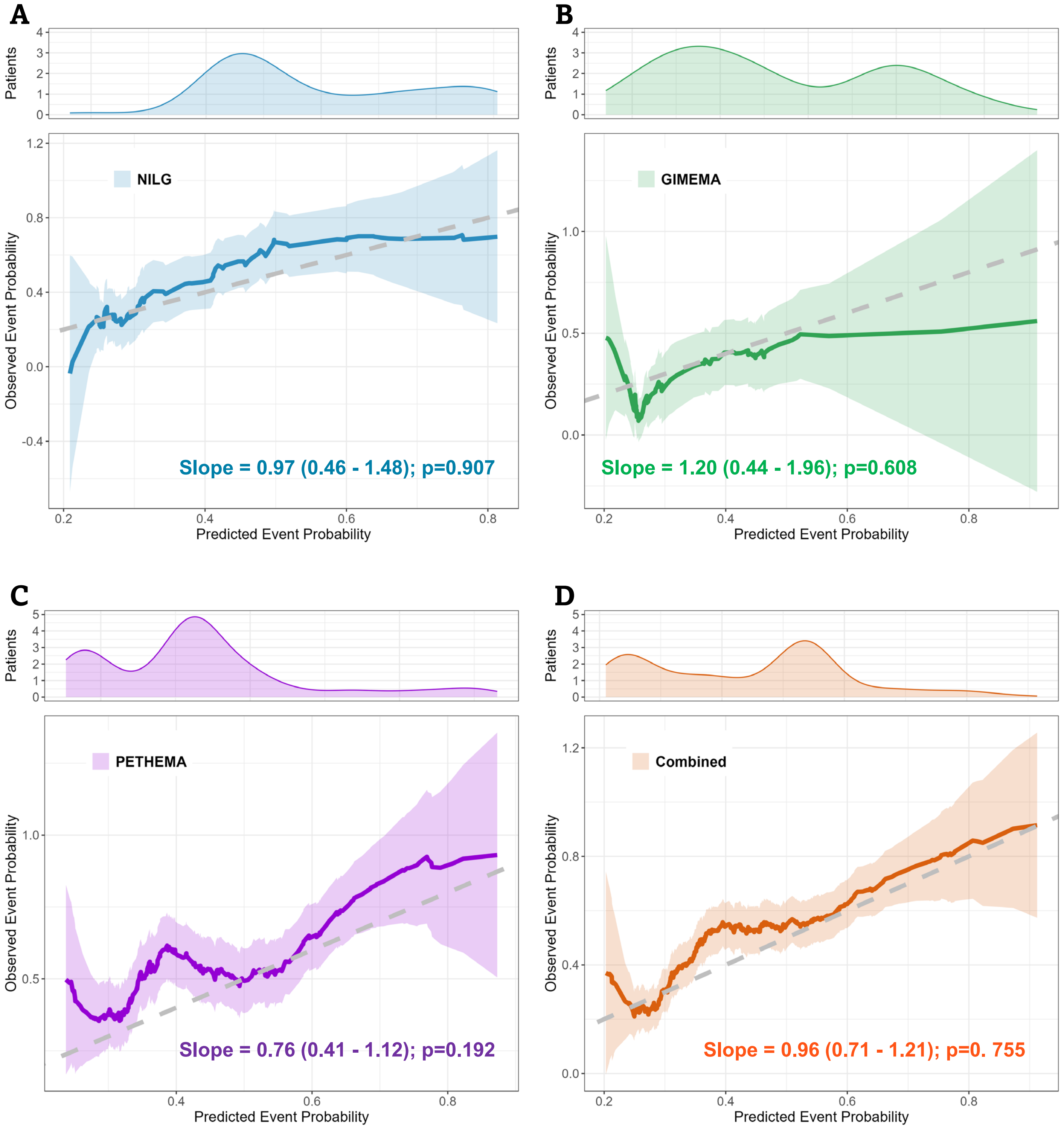

Supplementary Figure 2: Calibration plot for the EWALL prognostic index.
Each graph compares the predicted event probability (x axis) with the observed event probability (y axis) for each trial dataset (A – NILG-ALL10/07; B – GIMEMA-LAL1913; C – PETHEMA-ALLHR2011; D - Combined) using UKALL14 as the baseline. The dotted grey line represents perfect calibration (i.e. 1) whereas the solid coloured line represents the actual calibration. The shaded area represents the 95% confidence interval. Above each graph is a density plot showing the number of patients at the event probability.

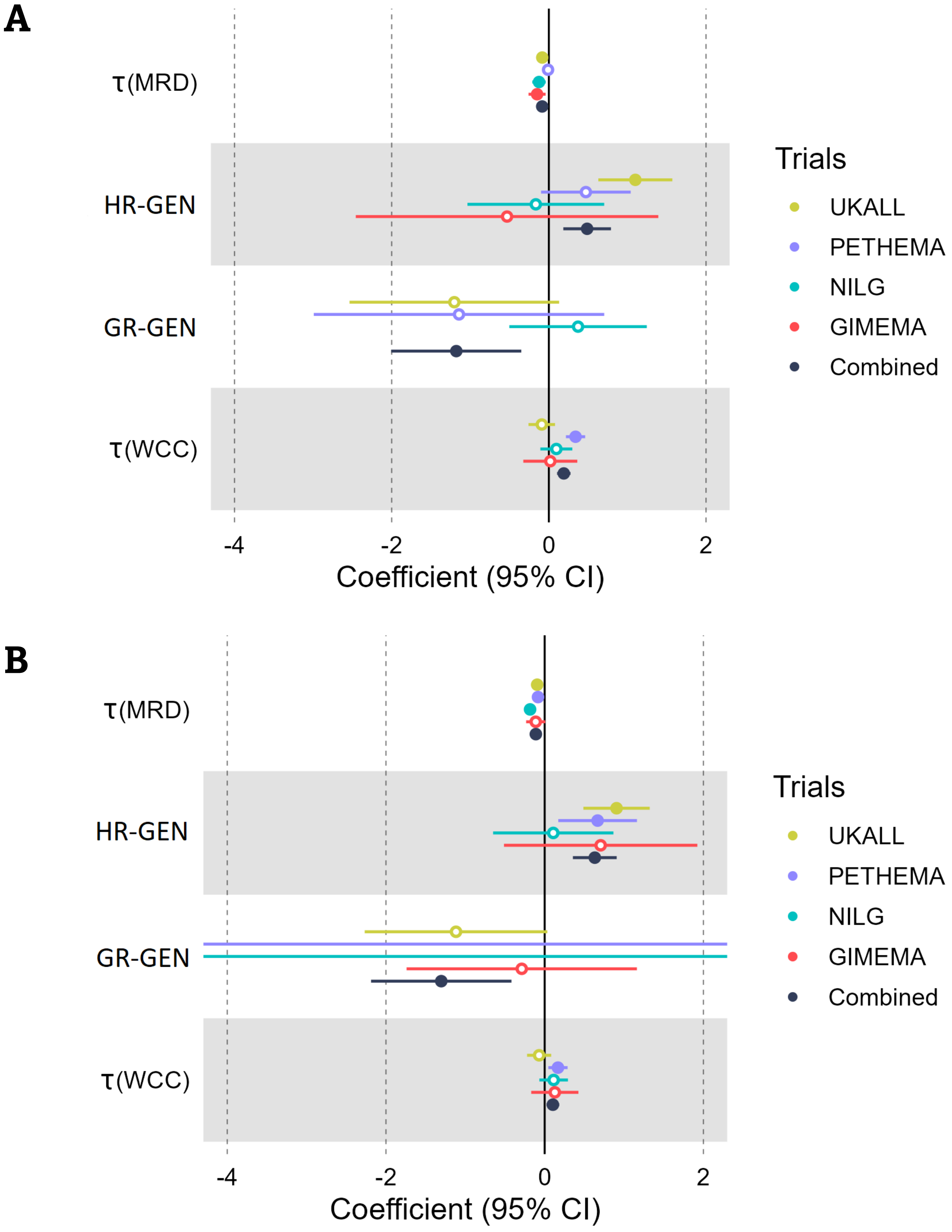

Supplementary Figure 3: Forest plot showing the coefficient and 95% confidence interval for relapse (A) derived from Fine-Gray competing risk model and death (B) derived from Cox regression analysis for each element of the EWALL prognostic index within each of the 4 trials and using a combined dataset. To explore whether the different components of the EWALL-PI contributed to the risk score equally in the different trials, we examined the coefficients from the Fine-Gray competing risk model (for relapse) and Cox regression model (for death) for each element within the EWALL-PI per study. The coefficients for minimal residual disease (MRD) and white blood cell count (WCC) were similar across the four studies indicating a high level of robustness: standard deviation was <0.05 for MRD and <0.16 for WCC for both Risk of Relapse (RR) and Overall Survival (OS). There was more variation for both HR-GEN (standard deviation of 0.62 for RR and 0.30 for OS) reflecting the completeness of genetic screening and for GR-GEN (standard deviation of 18.81 for RR and 16·43 for OS) reflecting their rarity in adult ALL.

Note: Coefficient of relapse, for GR-GEN in GIMEMA-LAL1913 is -44.08 and hence cannot be displayed in figure (A) as the x-axis is limited to -4 to 2. Abbreviations: HR-GEN - high-risk genetic abnormalities; GR-GEN - good-risk genetic abnormalities; CI - confidence interval.

Variables	Total Patients	Relapse	Death
**Sex**			
Male	152	40	54
	63	29	30
	63	11	14
	189	65	67
	467	145	165
Female	100	31	38
	46	14	19
	45	7	9
	119	47	42
	310	99	108
**Age (in years)**			
<25	0	0	0
	29	12	13
	33	3	4
	54	27	20
	116	42	37
25-39	117	25	32
	31	10	9
	32	5	7
	107	38	31
	287	78	79
40+	136	47	61
	49	21	27
	43	10	12
	147	47	58
	375	125	158
**White Cell Count (10^9/L)**			
<30	185	60	72
	71	28	29
	84	14	17
	186	49	56
	526	151	174
30+	68	12	21
	38	15	20
	24	4	6
	122	63	53
	252	94	100
**Immunophenotype**			
B-ALL	184	61	77
	73	30	37
	81	12	18
	209	72	76
	547	175	208
T-ALL	69	11	16
	36	13	12
	27	6	5
	99	40	33
	231	70	66

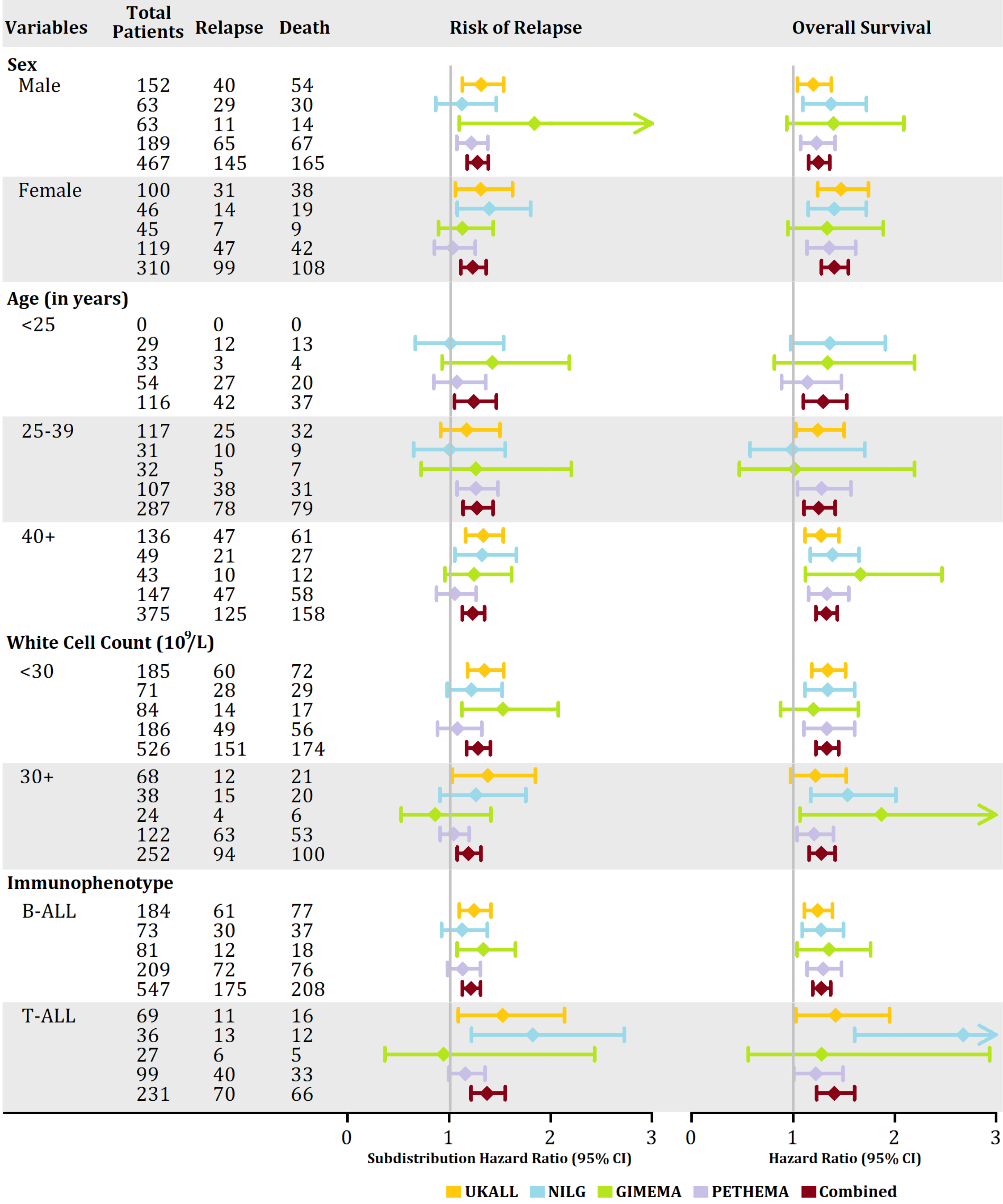

Supplementary Figure 4: Forest plots comparing subdistribution hazard ratios for risk of relapse derived from Fine-Gray competing risk model and hazard ratios for death derived from Cox regression analysis across the four different trials individually and using a combined dataset.

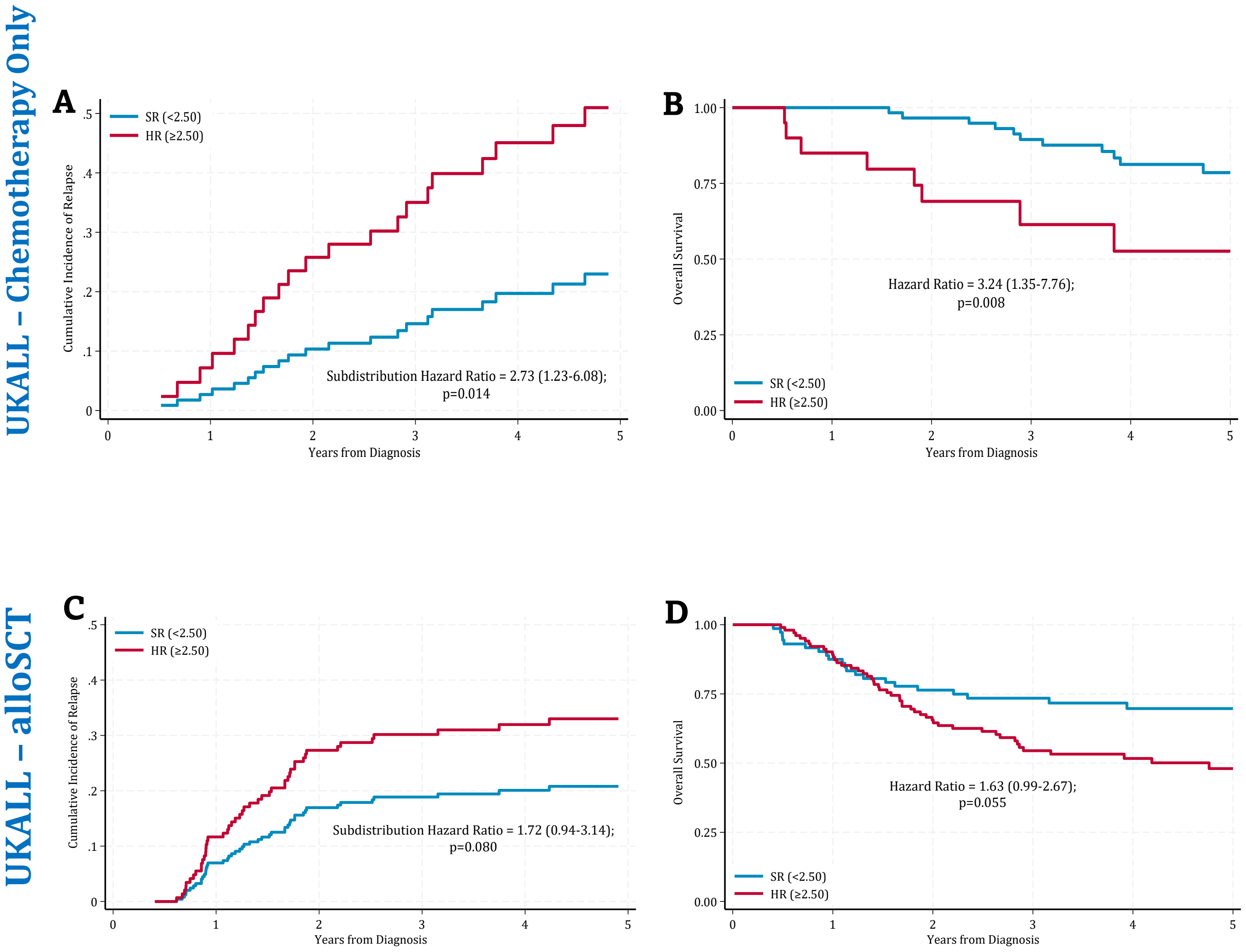

Supplementary Figure 5: Validation of EWALL-PI defined risk groups in UKALL14 patients who received chemotherapy only (A, B) and UKALL14 alloSCT patients (C, D).

Panels A and C show Kaplan-Meier plots for cumulative incidence of relapse (CIR) and the subdistribution hazard ratio for increased risk of relapse for HR patients versus SR patients. Panels B and D show the hazard ratio for overall survival (OS). Patients with a EWALL-PI <2·50 were assigned to the standard risk (SR) group (blue lines) whereas high risk (HR) patients had EWALL-PI ≥2·50 (red lines). **Abbreviation**: alloSCT, allogeneic stem cell transplant.

Supplementary Table 1: Details of the four contempoary adult ALL trials used in the study

**Trial Name**	**UKALL14**	**NILG ALL10/07**	**GIMEMA LAL1913**	**PETHEMA ALL-HR 2011**
**ClinicalTrials.gov NCT Number**	**01085617**	**00795756**	**02067143**	**01540812**
**Phase**	**3**	**2/3**	**2**	**Observational**
**Recruitment details**				
Dates	December 2010 - July 2018	January 2008 - October 2013	May 2014 - December 2020	August 2011 - October 2019
Age: Median (range)	41 (25 - 65)	41 (17–67)	34 (18 - 65)	40 (15 - 60)
**Total cases recruited to the trial**	827	203	203	403
Not eligible for EWALL- PI study				
Not ALL	13	0	20	38
No consent	3	0	0	0
BCR::ABL1 positive	199	42	0	4
Did not achieve complete remission	67	21	18 ^	15
Off protocol	107	0	8	1
Other reasons	0	0	10 #	23 *
Eligible	451	140	165	322
No MRD result	198	31	57	13
No WCC count	0	0	0	1
Cases included in the study	253	109	108	308
**Induction treatment**				
Number of phases	2	2	2	2
Total length	8 weeks	Week 10	Week 10 Ŧ	Week 5
Drugs	Induction 1: Daunorubicin, Vincristine, Dexamethasone, Pegylated Asparginase, Methotrexate. Induction 2: Cyclophosphamide, Cytarabine, Mercaptopurine, Methotrexate	Idarubicin, Vincristine, Dexamethasone, L-asparginase, IT prophylaxis	Induction 1: Cyclophosphamide; Prednisone (Prephase); Idarubicin, Vincristine, Dexamethasone, Pegylated Asparaginase. Induction 2: Idarubicin, Cyclophosphamide, Cytarabine, Mercaptopurine, Dexamethasone; ŦCycle 3: Methotrexate, Cytarabine.	Vincristine, Daunorubicin, Prednisone, Asparaginase (E. coli or pegylated), FLAG-Ida (fludarabine, cytarabine, granulocyte-colony-stimulating factor, and idarubicin)
**Minimal Residual Disease (MRD)**				
Methodology	Ig/TCR PCR	Ig/TCR PCR	Ig/TCR PCR	Flow cytometry
Timepoints	Week 4, 8	Week 10, 16, 22	Week 4, 10	Week 5, 16
Post-induction decision timepoint	Week 8	Week 10	Week 10-22	Week 5
**Risk stratification criteria**				
Standard risk	All other cases	BCP-ALL: WCC count <$30x10^9$/l, non-pro-B phenotype, no BCR::ABL1; T-ALL WCC count <$100x10^9$/l plus the cortical CD1a+ phenotype in T-ALL.	SR patients displayed no risk features.	All other cases
High risk	*BCR::ABL1* , *KMT2A::AFF1* , low hypodiploidy (30-39 chromosomes), complex karyotype (5 or more chromosomal abnormalities), presenting WCC count >$30x10^9$/l(B) or >100 (T), MRD (>0.01%) at the end of the second phase of induction or age >40y	All other cases	WCC count >30 and <100 $x10^9$/l (B-ALL only), a pro-B phenotype, or achieved a late CR after chemotherapy cycle 2.	No CR or MRD≥0.1%
Very High Risk	Not applicable	WCC count >$100x10^9$/l, high risk genetics (*KTM2A::AFF1* , abnormal 11q23, +8, -7, del6q, t(8;14)(q24;q32), low hypodiploidy (30-39 chromosomes), or a complex karyotype with ≥5 unrelated anomalies), and early/ mature T-ALL		Not applicable
Proportion high-risk (including very high risk)¶	66 (26%)	17 (16%)	9 (8%)	37 (12%)
**Transplant eligibility criteria**				
Eligibility criteria	All patients classified as high-risk	All VHR patients who achieved CR plus SR/HR patients with TP2 MRD(≥0.01%) or MRD positive week 22/TP4, plus HR patients without MRD study.		Patients with CR plus EOI MRD ≥0.1% and patients with MRD ≥0.01% after early consolidation
Rate ¶	68%	48%	27%	23%
**Outcome at 3 years**				
Median follow-up time in years (IQR)	4·53 (3·42 - 6·19)	7·87 (7·30 - 9·79)	3·25 (2·60 - 3·73)	2·62 (1·19 - 4·68)
Cumulative Incidence of Relapse	23.7% (18.6 - 29.2)	30.3% (22.0 - 39.0)	16.4% (9.8 - 24.5)	41.9% (35.6 - 48.1)
Event free survival	62·5% (56·1 - 68·2)	58.7% (48.9 - 67.3)	72·6% (62·5 - 80·4)	47·2% (40·7 - 53·4)
Overall survival	68·6% (62·4 - 74·1)	66% (56.3 - 74.1)	77·2% (67·3 - 84·5)	62·3% (55·8 - 68·2)

Key: ¶ for cases included in the study; ^including 8 off-protocol and 10 early deaths; #early deaths; Ŧincludes cycle 3, after which comes the mrd TP used; * death in induction (n = 15), age >60 years (n = 6), absence of HR criteria (n = 1), severe psychiatric disorder (n = 1).

Supplemenatry Table 2: Demographic, clinical, genetic and outcome features for patients treated on UKALL14, NILG ALL10/07, GIMEMA LAL1913, PETHEMA ALL-HR 2011 eligible for this study stratified by whether or not the prognostic index (PI) could be calculated

Variables	UKALL14				NILG ALL10/07				GIMEMA LAL1913				PETHEMA ALL-HR2011				p value comparig PI cohort across the four trials
	Eligible	PI	No PI	p value*	Eligible	PI	No PI	p value*	Eligible	PI	No PI	p value*	Eligible	PI	No PI	p value*	
**Total cases**	**451**	**253**	**198**		**140**	**109**	**31**		**165**	**108**	**57**		**322**	**308**	**14**		
**Sex**																	
Female	181 (40·1)	100 (39·5)	81 (41)	1·00	61 (44)	46 (42)	15 (48)	0·67	74 (45)	45 (42)	29 (51)	0·53	124 (39)	119 (39)	5 (36)	1·00	0·89
Male	269 (59·7)	152 (60·1)	117 (59)	1·00	79 (56)	63 (58)	16 (52)	0·67	91 (55)	63 (58)	28 (49)	0·53	198 (61)	189 (61)	9 (64)	1·00	
Intersex	1 (0·2)	1 (0·4)	··	0·48	··	··	··	··	··	··	··	··	··	··	··	··	
**Age (in years)**																	
<25	··	··	··	··	35 (25)	29 (27)	6 (19)	0·63	38 (23)	33 (31)	5 (9)	**0·05**	58 (18)	54 (18)	4 (29)	1·00	**<0·001**
25-39	201 (45)	117 (46)	84 (42)	0·75	40 (29)	31 (28)	9 (29)	0·82	45 (27)	32 (30)	13 (23)	0·48	110 (34)	107 (35)	3 (21)	0·71	
40-59	222 (49)	117 (46)	105 (53)	0·34	53 (38)	42 (39)	11 (35)	0·83	63 (38)	33 (31)	30 (53)	0·13	149 (46)	143 (46)	6 (43)	1·00	
60+	28 (6)	19 (8)	9 (5)	0·18	12 (9)	7 (6)	5 (16)	0·27	19 (12)	10 (9)	9 (16)	0·34	5 (2)	4 (1)	1 (7)	0·21	
**Immunophenotype**																	
B Cell	334 (74)	184 (73)	150 (76)	0·72	97 (69)	73 (67)	24 (77)	0·65	120 (73)	81 (75)	39 (68)	0·64	218 (68)	209 (68)	9 (64)	1·00	0·35
T Cell	117 (26)	69 (27)	48 (24)	0·72	43 (31)	36 (33)	7 (23)	0·65	45 (27)	27 (25)	18 (32)	0·64	104 (32)	99 (32)	5 (36)	1·00	
**White blood cell count (10^9/L)**																	
<30	341 (76)	185 (73)	156 (79)	0·26	99 (71)	71 (65)	28 (90)	0·17	128 (78)	84 (78)	44 (77)	1·00	194 (60)	186 (60)	8 (62)	1·00	**0·02**
30-49	31 (7)	19 (8)	12 (6)	0·53	9 (6)	9 (8)	··	0·38	11 (7)	6 (6)	5 (9)	0·68	31 (10)	30 (10)	1 (8)	1·00	
50-99	33 (7)	22 (9)	11 (6)	0·21	9 (6)	9 (8)	··	0·38	14 (8)	9 (8)	5 (9)	1·00	33 (10)	32 (10)	1 (8)	1·00	
≥100	46 (10)	27 (11)	19 (10)	0·60	23 (16)	20 (18)	3 (10)	0·57	12 (7)	9 (8)	3 (5)	0·68	63 (20)	60 (19)	3 (23)	0·66	
**Genetic risk group**																	
Good	26 (6)	19 (8)	7 (4)	0·18	4 (3)	3 (3)	1 (3)	1·00	13 (8)	11 (10)	2 (4)	0·44	10 (3)	9 (3)	1 (7)	1·00	**0·01**
High	118 (26)	66 (26)	52 (26)	1·00	25 (18)	17 (16)	8 (26)	0·59	25 (15)	9 (8)	16 (28)	**0·04**	38 (12)	37 (12)	1 (7)	1·00	**<0·001**
**Genetic Subgroup**																	
Good risk																	
ETV6::RUNX1	2 (0)	2 (1)	··	0·31	··	··	··	··	··	··	··	··	2 (1)	1 (0)	1 (7)	0·50	NC
High hyperdiploidy	13 (3)	7 (3)	6 (3)	0·93	4 (3)	3 (3)	1 (3)	0·95	7 (4)	6 (6)	1 (2)	0·47	7 (2)	7 (2)	··	0·91	NC
ZNF384	11 (2)	10 (4)	1 (1)	0·10	··	··	··	··	6 (4)	5 (5)	1 (2)	0·58	1 (0)	1 (0)	··	1·00	NC
High risk																	
KMT2A	36 (8)	19 (8)	17 (9)	0·77	12 (9)	9 (8)	3 (10)	0·91	14 (8)	··	14 (25)	··	22 (7)	21 (7)	1 (7)	1·00	NC
JAK-STAT	24 (5)	16 (6)	8 (4)	0·48	2 (1)	1 (1)	1 (3)	0·66	7 (4)	5 (5)	2 (4)	0·84	··	··	··	··	NC
Low hypodiploidy	31 (7)	18 (7)	13 (7)	0·90	3 (2)	2 (2)	1 (3)	0·83	1 (1)	1 (1)	··	0·69	7 (2)	7 (2)	··	0·91	NC
iAMP21	4 (1)	1 (0)	3 (2)	0·40	··	··	··	··	··	··	··	··	··	··	··	-	NC
Complex karyotype	23 (5)	12 (5)	11 (6)	0·77	8 (6)	5 (5)	3 (10)	0·62	3 (2)	3 (3)	··	0·43	9 (3)	9 (3)	··	0·92	NC
Intermedate risk																	
TCF3::PBX1	12 (3)	11 (4)	1 (1)	0·12	3 (2)	3 (3)	··	0·61	5 (3)	··	5 (9)	··	··	··	··	··	NC
ABL-class	8 (2)	7 (3)	1 (1)	0·23	··	··	··	··	··	··	··	··	··	··	··	··	NC
Other	191 (42)	109 (43)	82 (41)	0·82	108 (77)	86 (79)	22 (71)	0·66	122 (74)	88 (81)	34 (60)	0·07	185 (57)	178 (58)	7 (50)	0·92	NC
Failed/No data	96 (21)	41 (16)	55 (28)	**0·05**	··	··	··	··	··	··	··	··	89 (28)	84 (27)	5 (36)	0·91	NC
**Trial risk group**																	
Standard	129 (29)	64 (25)	65 (33)	0·16	55 (39)	41 (38)	14 (45)	0·83	88 (53)	66 (61)	22 (39)	0·10	225 (70)	223 (72)	2 (14)	0·44	**<0·001**
High	322 (71)	189 (75)	133 (67)	0·16	85 (61)	68 (62)	17 (55)	0·83	77 (47)	42 (39)	35 (61)	0·10	94 (29)	85 (28)	9 (64)	0·70	
Not evaluable	··	··	··	··	··	··	··	··	··	··	··	··	3 (1)	··	3 (21)	··	
**Minimal Residual Disease at end of induction**																	
Negative (<0·01%)	191 (75)	191 (75)	··	··	77 (71)	77 (71)	··	··	85 (79)	85 (79)	··	··	187 (60)	185 (60)	··	··	**<0·001**
Positive (≥0·01%)	62 (25)	62 (25)	··	··	32 (29)	32 (29)	··	··	23 (21)	23 (21)	··	··	123 (40)	123 (40)	··	··	
**alloSCT in first complete remission**																	
Yes	294 (65)	173 (68)	121 (61)	0·32	65 (46)	52 (48)	13 (42)	0·68	52 (32)	29 (27)	23 (40)	0·27	76 (24)	70 (23)	6 (43)	0·68	**<0·001**
**Outcome**																	
Relapse	125 (28)	72 (28)	53 (27)		54 (39)	43 (39)	11 (35)		38 (23)	18 (17)	20 (35)		117 (36)	112 (36)	5 (36)		
Death	171 (38)	93 (37)	78 (39)		60 (43)	49 (45)	11 (35)		43 (26)	23 (21)	20 (35)		116 (36)	109 (35)	7 (50)		
CIR at 3 years (95% CI)	24% (20 - 28)	24% (19 - 29)	25% (19 - 31)	0·99	30% (23 - 38)	30% (22 - 39)	31% (15 - 48)	0·94	22% (16 - 29)	16% (10 - 25)	34% (21 - 46)	**0·001**	42% (36 - 48)	42% (36 - 48)	40% (14 - 65)	0·31	**<0·001**
OS at 3 years (95% CI)	67% (62 - 71)	69% (62 - 74)	64% (57 - 70)	0·37	68% (59 - 75)	66% (56 - 74)	76% (55 - 88)	0·61	73% (65 - 79)	77% (67 - 84)	64% (48 - 76)	0·01	61% (55 - 67)	62% (56 - 68)	46% (18 - 70)	0·10	**0·01**
Median follow-up (years) (IQR)	4·6 (3·4 - 6·1)	4·5 (3·4 - 6·2)	4·7 (3·1 - 6·1)	··	7·7 (6·8 - 9·5)	7·9 (7·3 - 9·8)	6·7 (4·7 - 8·1)	··	3·1 (2·5 - 3·6)	3·2 (2·6 - 3·7)	2·6 (2·1 - 3·3)	··	2·5 (1·2 - 4·6)	2·6 (1·2 - 4·7)	2·1 (1·7 - 3·0)	··	··

Key: *p value comparing the proportion of eligible patients vs patients with PI available. **Abbreviations:** PI - prognostic index; alloSCT - allogeneic stem cell transplant; CIR - cumulative incidence of relapse; OS - overall survival; CI - confidence interval; IQR - inter-quartile range; NC, not calculated - too few cases.

Supplementary Table 3: Subdistribution hazard ratios (SHR) for relapse risk and hazard ratios (HR) for overall survival with 95% confidence intervals (CI) showing the increased risk of relapse or death associated with each unit increase in the EWALL-PI.

	UKALL14 (n=253)		NILG-ALL10/07 (n=109)		GIMEMA-LAL1913 (n=108)		PETHEMA-ALL-HR2011 (n=308)	
	**Univariate**	**Age adjusted**	**Univariate**	**Age adjusted**	**Univariate**	**Age adjusted**	**Univariate**	**Age adjusted**
**Relapse Risk**								
EWALL-PI	1·30 (1·15-1·47), p<0·001	1·30 (1·15-1·47), p<0·001	1·23 (1·03-1·48), p=0·023	1·23 (1·03-1·47), p=0·025	1·29 (1·05-1·58), p=0·017	1·23 (0.99-1·53), p=0·066	1·15 (1·03-1·27), p=0·012	1·13 (1·02-1·26), p=0·023
Age	··	1·03 (1·01-1·05), p=0·01	··	1·01 (0·98-1·03), p=0·502	··	1·03 (1·00-1·05), p=0·069	··	0·98 (0·97-1·00), p=0·012
**Overall Survival**								
EWALL-PI	1·28 (1·15-1·42), p<0·001	1·26 (1·13-1·40), p<0·001	1·40 (1·21-1·62), p<0·001	1·38 (1·19-1·59), p<0·001	1·35 (1·05-1·75), p=0·02	1·39 (1·06-1·82), p=0·02	1·27 (1·14-1·42), p<0·001	1·29 (1·15-1·43), p<0·001
Age	··	1·03 (1·01-1·05), p<0·001	··	1·02 (1·00-1·04), p=0·08	··	1·04 (1·01-1·07), p=0·01	··	1·01 (1·00-1·03), p=0·16

* Age-adjusted using age as a continuous variable.

Supplementary Table 4: Harrell's Concordance Index and 95% confidence interval derived from Fine-Gray competing risk models for relapse and Cox regression models for overall survival using the EWALL PI and original definitions to predict outcome

Outcome measure/prognostic factor	UKALL14	NILG ALL10/07	GIMEMA LAL1913	PETHEMA ALL-HR 2011
**Relape Free Survival**				
EWALL-PI Continuous variable	0·65 (0·59 - 0·72)	0·64 (0·55 - 0·74)	0·69 (0·56 - 0·82)	0·60 (0·54 - 0·65)
EWALL-PI Defined risk groups (SR<2·50; HR≥2·50)	0·60 (0·55 - 0·66)	0·61 (0·54 - 0·69)	0·63 (0·51 - 0·75)	0·57 (0·54 - 0·61)
Original risk groups defined by trial protocol*	0·56 (0·52 - 0·61)	0·57 (0·50 - 0·64)	0·51 (0·39 - 0·63)	0·50 (0·45 - 0·54)
alloSCT (received Yes/No)	0·54 (0·49 - 0·59)	0·64 (0·57 - 0·70)	0·58 (0·50 - 0·66)	0·56 (0·53 - 0·60)
**Overall Survival**				
EWALL-PI Continuous variable	0·64 (0·58 - 0·70)	0·67 (0·58 - 0·76)	0·59 (0·45 - 0·74)	0·64 (0·58 - 0·69)
EWALL-PI Defined risk groups (SR<2·50; HR≥2·50)	0·60 (0·55 - 0·65)	0·64 (0·58 - 0·71)	0·57 (0·47 - 0·68)	0·56 (0·52 - 0·60)
Original risk groups defined by trial protocol*	0·57 (0·53 - 0·61)	0·57 (0·50 - 0·64)	0·57 (0·46 - 0·67)	0·58 (0·54 - 0·63)
alloSCT (received Yes/No)	0·58 (0·54 - 0·63)	0·57 (0·50 - 0·65)	0·58 (0·50 - 0·65)	0·49 (0·45 - 0·52)

Key: * The following risk groupings were used when calculating Harrell's C-index: UKALL14 (SR v HR), NILG-ALL10/07 (SR v HR/VHR), GIMEMA-LAL1913 (SR v HR/VHR) and PETHEMA-ALL-HR2011 (SR v HR). **Abbreviations:** SR - standard risk; HR - high risk; VHR - very high risk; alloSCT - allogeneic stem cell transplant.

Supplementary Table 5: Demographic, clinical, genetic and outcome features of patients treated on UKALL14 eligible for this study, stratified by post-induction therapy

Variable	Total	Chemotherapy Only	alloSCT	p value*
**Total patients**	253	79	174	
**Sex**				**0·03**
Female	100 (39·5)	38 (48)	62 (36)	
Male	152 (60·1)	40 (51)	112 (64)	
Intersex	1 (0·4)	1 (1)	0 (0)	
**Age (in years)**				**<0·001**
25-39	117 (46)	54 (68)	63 (36)	
40-59	117 (46)	18 (23)	99 (57)	
60+	19 (8)	7 (9)	12 (7)	
**Immunophenotype**				0·87
B Cell	184 (73)	58 (73)	126 (72)	
T Cell	69 (27)	21 (27)	48 (28)	
**White blood cell count (10^9/L)**				0·12
<30	185 (73)	60 (76)	125 (72)	
30-49	19 (8)	5 (6)	14 (8)	
50-99	22 (9)	10 (13)	12 (7)	
≥100	27 (11)	4 (5)	23 (13)	
**Genetic risk group**				
Good	19 (8)	11 (14)	8 (5)	**0·01**
High	66 (26)	10 (13)	56 (32)	**0·001**
**Genetic Subgroup**				
Good risk				
ETV6::RUNX1	2 (1)	2 (3)	··	
High hyperdiploidy	7 (3)	3 (4)	4 (2)	
ZNF384	10 (4)	6 (8)	4 (2)	
High risk				
KMT2A	19 (8)	2 (3)	17 (10)	
JAK-STAT	16 (6)	3 (4)	13 (7)	
Low hypodiploidy	18 (7)	2 (3)	16 (9)	
iAMP21	1 (0)	1 (1)	··	
Complex karyotype	12 (5)	2 (3)	10 (6)	
Intermediate risk				
TCF3::PBX1	11 (4)	6 (8)	5 (3)	
ABL-class	7 (3)	2 (3)	5 (3)	
Other	109 (43)	34 (43)	75 (43)	
Failed/No data	41 (16)	16 (20)	25 (14)	
**Trial risk group**				**<0·001**
Standard	64 (25)	45 (57)	19 (11)	
High	189 (75)	34 (43)	155 (89)	
**Minimal Residual Disease at end of induction**				**<0·001**
Negative (<0·01%)	191 (75)	76 (96)	115 (66)	
Positive (≥0·01%)	62 (25)	3 (4)	59 (34)	
**Outcome**				
Relapse	72 (28)	24 (30)	48 (28)	
Dead	93 (37)	21 (27)	72 (41)	
CIR at 3 years (95% CI)	24% (19 - 29)	20% (12 - 29)	26% (19 - 32)	0.596
OS at 3 years (95% CI)	69% (62 - 74)	83% (72 - 90)	62% (54 - 69)	**0.008**
Median follow-up (years) (IQR)	4·5 (3·4 - 6·2)	4·9 (3·7 - 6·4)	4·5 (3·4 - 5·9)	
**Prognostic Index: Median (range)**	2·4 (0·5 - 8·0)	1·8 (0·5 - 7·3)	3·2 (0·5 - 8·0)	**<0·001**
**PI defined risk group**				**<0·001**
Standard (<2·50)	131 (52)	59 (75)	72 (41)	
High (≥2·50)	122 (48)	20 (25)	102 (59)	

Key: *P value comparing chemotherapy only vs alloSCT patients. **Abbreviations:** PI - prognostic index; CIR - cumulative incidence of relapse; OS - Overall survival; CI - confidence interval; IQR - inter-quartile range; alloSCT - allogeneic stem cell transplant.

Supplementary Table 7: Distribution and outcome of trial patients classified according to the original risk groups, post-induction therapy and by EWALL-PI defined risk groups

Original Trial Risk Groups / Post-inudction therapy received	EWALL-PI defined risk groups*											
	UKALL14			NILG ALL10/07			GIMEMA LAL1913			PETHEMA ALL-HR 2011		
	SR	HR	Total	SR	HR	Total	SR	HR	Total	SR	HR	Total
**Number of patients**												
**SR**	54	10	64	38	3	41	46	20	66	68	155	223
**HR**	77	112	189	25	43	68	28	14	42	0	85	85
**Total**	131	122	253	63	46	109	74	34	108	68	240	308
**Cumulative Incidence of Relapse at 3 years, % (95% CI)**												
**SR**	15% (7 - 26)	10% (1 - 36)	14% (7 - 24)	18% (8 - 32)	33% (1 - 77)	20% (9 - 33)	9% (3 - 20)	41% (18 - 64)	18% (9 - 28)	28% (16 - 42)	50% (40 - 58)	43% (35 - 50)
**HR**	17% (10 - 26)	34% (25 - 42)	27% (21 - 33)	20% (7 - 37)	47% (31 - 60)	37% (26 - 48)	15% (5 - 31)	8% (1 - 31)	13% (5 - 26)	··	39% (28 - 50)	39% (28 - 50)
**Total**	16% (11 - 23)	32% (24 - 40)	··	19% (11 - 30)	46% (31 - 59)	··	12% (5 - 20)	28% (13 - 46)	··	28% (16 - 42)	46% (39 - 53)	··
**Overall Survival at 3 years, % (95% CI)**												
**SR**	85% (72 - 92)	77% (34 - 94)	84% (72 - 91)	79% (62 - 89)	100%	80% (65 - 90)	86% (72 - 94)	68% (41 - 84)	81% (68 - 88)	80% (65 - 88)	64% (55 - 72)	69% (61 - 75)
**HR**	77% (66 - 85)	54% (44 - 63)	63% (56 - 70)	80% (58 - 91)	44% (29 - 58)	57% (45 - 68)	70% (45 - 85)	76% (42 - 91)	72% (53 - 84)	··	46% (34 - 57)	46% (34 - 57)
**Total**	80% (72 - 86)	56% (46 - 64)	··	79% (67 - 87)	48% (33 - 61)	··	80% (68 - 88)	71% (51 - 84)	··	80% (65 - 88)	57% (50 - 64)	··
**Number of patients**												
**Chemotherapy only**	59	20	79	40	17	57	57	22	79	64	174	238
**alloSCT**	72	102	174	23	29	52	17	12	29	4	66	70
**Total**	131	122	253	63	46	109	74	34	108	68	240	308
**Cumulative Incidence of Relapse at 3 years, % (95% CI)**												
**Chemotherapy only**	15% (8 - 26)	31% (13 - 52)	19% (11 - 29)	28% (15 - 42)	76% (49 - 90)	42% (29 - 54)	11% (4 - 21)	44% (21 - 65)	19% (11 - 29)	29% (17 - 43)	51% (42 - 59)	45% (37 - 52)
**alloSCT**	17% (9 - 27)	32% (23 - 41)	26% (19 - 32)	4% (0 - 18)	28% (13 - 44)	17% (9 - 29)	13% (2 - 33)	0%	7% (1 - 21)	0%	34% (22 - 47)	33% (21 - 46)
**Total**	16% (11 - 23)	32% (24 - 40)	··	19% (11 - 30)	46% (31 - 59)	··	12% (5 - 20)	28% (13 - 46)	··	28% (16 - 42)	46% (39 - 53)	··
**Overall Survival at 3 years, % (95% CI)**												
**Chemotherapy only**	90% (78 - 95)	61% (35 - 80)	83% (73 - 90)	75% (59 - 86)	24% (7 - 45)	60% (46 - 71)	81% (67 - 89)	53% (29 - 72)	73% (61 - 82)	79% (64 - 88)	58% (50 - 66)	64% (57 - 71)
**alloSCT**	73% (61 - 82)	54% (44 - 64)	62% (54 - 69)	87% (65 - 96)	62% (42 - 77)	73% (59 - 83)	81% (52 - 94)	100%	89% (70 - 96)	100%	56% (41 - 68)	57% (42 - 69)
**Total**	80% (72 - 86)	56% (46 - 64)	··	79% (67 - 87)	48% (33 - 61)	··	80% (68 - 88)	71% (51 - 84)	··	80% (65 - 88)	57% (50 - 64)	··

Abbreviations: SR - standard risk; HR - high risk; alloSCT - allogeneic stem cell transplant.

Supplementary Table 6: Demographic, clinical, genetic and outcome features of patients treated on UKALL14 eligible for this study, assigned to the EWALL-PI defined risk groups, among post-induction therapy groups

Variables	UKALL14 - Chemotherapy Only Patients			UKALL14 - alloSCT Patients		
	SR	HR	P value	SR	HR	P value
**Total cases**	**59**	**20**		**72**	**102**	
**Sex**						
Female	29 (49)	9 (45)		23 (32)	39 (38)	
Male	29 (49)	11 (55)	0·85	49 (68)	63 (62)	0·39
Intersex	1 (2)	0 (0)				
**Age (years): Median (range)**	32 (25 - 63)	39 (25 - 63)	0·06	44 (25 - 65)	44 (25 - 63)	0·88
<25	-	-		-	-	
25-39	43 (73)	11 (55)	**0·03**	25 (35)	38 (37)	0·75
40-59	14 (24)	4 (20)		43 (60)	56 (55)	
60+	2 (3)	5 (25)		4 (6)	8 (8)	
**WCC (10^9/L): Median (range)**	7.8 (0.5 - 254.6)	17.6 (0.4 - 168.6)	0·15	8.3 (0.1 - 338.8)	12.0 (0.4 - 583.1)	0·13
<30	45 (76)	15 (75)		55 (76)	70 (69)	
30-49	4 (7)	1 (5)	0·71	3 (4)	11 (11)	0·44
50-99	8 (14)	2 (10)		5 (7)	7 (7)	
≥100	2 (3)	2 (10)		9 (12)	14 (14)	
**Immunophenotype**						
B-cell	43 (73)	15 (75)	0·85	45 (62)	81 (79)	**0·01**
T-cell	16 (27)	5 (25)		27 (38)	21 (21)	
**Minimal Residual Disease**						
Negative (<0·01%)	59 (100)	17 (85)	**0·01**	72 (100)	43 (42)	**<0·001**
Positive (≥0·01%)	0 (0)	3 (15)		0 (0)	59 (58)	
**Genetic Risk group**						
Good	9 (15)	2 (10)	0·72	4 (6)	4 (4)	0·72
High	0 (0)	10 (50)	**<0·001**	0 (0)	56 (55)	**<0·001**
**Outcome**						
Relapse	14 (24)	10 (50)		15 (21)	33 (32)	
Death	12 (20)	9 (45)		23 (32)	49 (48)	
alloSCT	-	-	-	72 (100)	102 (100)	-
**Survival Rates at 3 years**						
Cumulative Incidence of Relapse	16% (8 - 26)	31% (13 - 52)	**0·005**	17% (9 - 26)	32% (23 - 41)	0·07
Overall survival	89% (78 - 95)	61% (35 - 80)	**0·001**	73% (62 - 82)	54% (44 - 64)	0·05

Abbreviations: SR - standard risk; HR - high risk; WCC - white blood cell count; alloSCT - allogeneic stem cell transplant.

Calculate the EWALL Prognostic Index (PI)

Newcastle University

**Minimal Residual Disease**	1.06E-05	
Absolute MRD value from end of induction. If positive outside the quantitative range input 0·00001.	**Correct**	
**Genetic Good Risk**	0	Please enter 0 or 1 only
Does the patient have a good risk abnormality **(1)**- *ETV6::RUNX1*, high hyperdiploidy, *ZNF384* rearrangements- or not **(0)**.	**Correct**	
**Genetic High Risk**	0	Please enter 0 or 1 only
Does the patient have a high risk abnormality **(1)**- *KMT2A* fusions, low hypodiploidy, near-haploidy, complex karyotype, JAK-STAT abnormalities (*IGH::CRLF2*, *P2RY8::CRLF2*, *JAK2* fusions), iAMP21- or not **(0)**.	**Correct**	
**White Blood Cell Count (10^9/L)**	2.5	
Pre treatment white blood cell count.	**Correct**	
**Prognostic Index Value:**	**2.294**	
**This PI would place the patient in category:**	SR	

Disclaimer- this spreadsheet is for research purposes only. It is not intended for clinical use.
